# Patterns of digital health access and use among US adults: A latent class analysis.

**DOI:** 10.21203/rs.3.rs-3895228/v1

**Published:** 2024-01-29

**Authors:** Phillip Hegeman, Daniel Vader, Kristyn Kamke, Sherine El-Toukhy

## Abstract

Background Digital technologies allow users to engage in health-related behaviors associated with positive outcomes. We aimed to identify classes of US adults with distinct digital technologies access and health use patterns and characterize class composition. Data came from Health Information National Trends Survey Wave 5 Cycles 1–4, a nationally representative cross-sectional survey of US adults ( *N* = 13,993). We used latent class analysis to identify digital technologies access and health use patterns based on 32 behaviors and access to requisite technologies and platforms that include the internet, internet-enabled devices, health monitors, and electronic health records (EHRs). We ran a multinomial logistic regression to identify sociodemographic and health correlates of class membership ( *n* = 10,734). Results Ten classes captured patterns of digital technology access and health use among US adults. This included a digitally isolated, a mobile-dependent, and a super user class, which made up 8.9%, 7.8%, and 13.6% of US adults, respectively, and captured access patterns from only basic cellphones and health monitors to near complete access to web-, mobile-, and EHR-based platforms. Half of US adults belonged to classes that lacked access to EHRs and relied on alternative web-based tools typical of patient portals. The proportion of class members who used digital technologies for health purposes varied from small to large. Older and less educated adults had lower odds of belonging to classes characterized by access or engagement in health behaviors. Hispanic and Asian adults had higher odds of belonging to the mobile-dependent class. Individuals without a regular healthcare provider and those who had not visited a provider in the past year were more likely to belong to classes with limited digital technologies access or health use. Discussion Only one third of US adults belonged to classes that had near complete access to digital technologies and whose members engaged in almost all health behaviors examined. Sex, age, and education were associated with membership in classes that lacked access to 1 + digital technologies or exhibited none to limited health uses of such technologies. Results can guide efforts to improve access and health use of digital technologies to maximize associated health benefits and minimize disparities.

### Introduction

Digital technologies allow users to engage in various health-supporting activities.[1] In 2018, 70.1% of US adults looked up health information online.[2] They are increasingly using mobile devices (38.9%) and wellness and medical wearables (35.3%) to track their health, and 17.2% share self-generated data with healthcare professionals.[2] In 2020, 39.5% of US adults accessed their electronic medical records.[3] Use of digital technologies is broadly associated with positive health outcomes. For example, online health information seeking is associated with being informed, holding positive health attitudes, and adopting healthy behaviors.[4–7] Social media use is associated with increased access to health information and perceived social support.[8–10] Text messaging and app-based interventions are effective for behavior modification and health management.[11,12] Fitness and medical wearables are useful for health monitoring, detection, and prediction of health outcomes, which can improve medical decisions and patient outcomes.[13–17] Patients with access to their medical records make informed decisions, adhere to preventative behaviors and treatment regimens, are satisfied with care, and have better patient-physician relationships.[18–21]

Despite digital technology access and use being at an all-time high, disparities exist. In 2021, 77% of US adults had broadband internet at home[22] and 97% owned mobile phones, with smartphone ownership at 85% and basic cellphone ownership at 11%.[23] However, younger, more educated, and high-income earning adults are more likely to own tablets and smartphones.[23] A smaller percentage of Black and Hispanic adults own a laptop/desktop computer or have home broadband than Whites.[24] Younger, less educated, racial/ethnic minority, and low-income adults are also more likely to be smartphone-dependent for internet access.[23] Looking across multiple technologies, 63% of adults living in households earning ≥$100,000 annually have joint access to broadband internet, a computer, smartphone, and tablet compared to only 23% of adults in households earning <$30,000.[25]

Beyond access, sociodemographic disparities manifest in the use of digital technologies. For example, although 93% of US adults use the internet, only 75% of adults aged 65+, 86% of adults with high school education or less, and 86% of adults with income <$30,000 use the internet compared to ≥ 98% of adults ages 18–49, college graduates, and those with income ≥$50,000.[22] Despite a narrowing gap between urban/suburban and rural Americans' adoption of home broadband, rural residents go online less frequently than their urban counterparts.[26] Inequities also exist in use of specific technologies such as mobile health apps,[27,28] wearable devices,[29,30] and patient portals.[31–36]

Access to and use of digital technologies are prerequisites for reaping associated health benefits. Patterns of digital technology access and use are interconnected in nature, resulting in countless combinations that can impact health outcomes in both direction and magnitude whereby they can exacerbate inequalities or compound health benefits.[37,38] Prior studies on digital technology access and use have focused on either access *or* use patterns of individual technologies such as mobile health apps,[27] wearables,[30] and patient portals rather than considering access and use jointly.[31–33,39,40] Furthermore, prior studies generally define patterns of digital health technologies a priori,[41,42] potentially failing to identify nuanced and previously unconsidered patterns of technology access and use. When studies report on multiple technologies, which can provide multiple avenues for engaging in health behaviors, there is no differentiation between general use and health-related use of those technologies.[43,44] Finally, previous research often focuses on specialized population (e.g., adults with chronic illness,[40] elderly adults[33,44]) rather than nationally representative samples, raising uncertainties about the generalizability of their findings.

Using a nationally representative sample of US adults, we aim to identify (1) latent classes of adults based on their patterns of access to and health uses of digital technologies and (2) sociodemographic and health correlates of membership in these classes.

## Methods

**Data**. Data came from 13,993 US adults ≥ 18 years old who responded to the Health Information National Trends Survey (HINTS) Wave 5, Cycles 1 (2017) through 4 (2020), thereafter H5C1 through H5C4. HINTS is a nationally representative cross-sectional survey of US adults which oversamples areas with high concentrations of racial and ethnic minority populations to increase precision of estimates for minority subpopulations.[45] All surveys were distributed by mail and answered via paper-and-pencil except for H5C3 where web options were offered to certain participants to examine the effects of mixed-mode design on response rates and sample representativeness. To avoid introducing mode of data collection as a potential source of bias, we only included the paper-and-pencil responses of H5C3 and their corresponding sampling weights. Data were collected between 1/25/2017 and 6/15/2020.

**Measures.** We identified 11 digital technology access questions and 32 health use questions. Access questions covered accessing the internet, using a home computer to access the internet, having a basic cell phone or smart mobile device, using electronic health monitors (including medical and fitness devices), and having been offered access to electronic health records (EHRs). Health use questions spanned health behaviors that people can engage in on the web and social media (e.g., seeking health information online), mobile and wearable devices (e.g., downloading health and wellness apps), and EHRs (e.g., viewing test results).

For each health use question, we created a three-level indicator considering both access to prerequisite digital technologies and corresponding behavior, coded as: 2 = respondent had access to requisite technologies and engaged in behavior, 1 = respondent had access to requisite technologies but did not engage in behavior, and 0 = respondent did not have access to requisite technologies and did not engage in behavior (Supplementary Note 1). This coding scheme differentiated between one's *choice* to not engage in a behavior and one's *inability* to engage in that behavior due to lack of access to requisite technologies.

**Analysis.** We conducted a latent class analysis (LCA), a structural equation modeling analysis in which observed indicator variables are related to a discrete latent class variable. Using Vermunt's 3-step approach,[46] step 1 consisted of running an LCA ($N$ = 13,993) with 32 indicators and no sociodemographic covariates. We fit models with 1 to 20 classes, which were evaluated using statistical fit indices to select a model with a specified number of classes. The fit indices used were Akaike's information criterion (AIC), consistent AIC (CAIC), Bayesian information criterion (BIC), sample size-adjusted BIC (SABIC), model entropy, and the Vuong-Lo-Mendell-Rubin adjusted likelihood ratio test (VLMR-LRT).[47] In step 2, we estimated participant probabilities of membership to each latent class, the most likely class a participant belonged to, and the corresponding measurement error. In step 3, we re-fit the latent class model selected in step 1 while including a multinomial logistic regression of most likely class membership onto the sociodemographic (e.g., sex) and health (e.g., having a regular healthcare provider) covariates ($n$ = 10,734).

LCA was done in Mplus[48] via the MplusAutomation package (version 1.1.0) in R,[49] with steps two and three performed together using the R3STEP Mplus auxiliary setting.[50] Population estimates were calculated in R. Overall sampling weights were used in all analyses to account for HINTS complex survey design and produce nationally representative estimates. Jackknife replicate weights (50 sets of weights each year, 200 total) were used to calculate standard errors and confidence intervals for population estimates and regression odds ratios.[45,51] Full information maximum likelihood was used to handle missing data in latent class indicators where a survey response contributed to the LCA if data were available for at least one indicator. Only 33 responses were missing on all indicators and were excluded from the initial LCA. For the multinomial logistic regression, listwise deletion was used, which resulted in the exclusion of ~ 22% of the analytic sample due to covariate missingness (Supplementary Note 1).

## Results

Weighted sociodemographic sample characteristics appear in Table 1.

Table 1
Weighted sample characteristics, HINTS 5, cycles 1 through 4, 2017–2020, $N$ = 13,993

| | H5C1 ($n$ = 3,287) | H5C2 ($n$ = 3,498) | H5C3* ($n$ = 3,349) | H5C4 ($n$ = 3,864) | Total ($N$ = 13,993) |
|---|---|---|---|---|---|
| | **% (95% CI)** | **% (95% CI)** | **% (95% CI)** | **% (95% CI)** | **% (95% CI)** |
| **Sex** | | | | | |
| Male | 48.09 (47.81, 48.38) | 48.12 (47.75, 48.50) | 48.12 (47.53, 48.70) | 47.57 (46.93, 48.22) | 47.98 (47.73, 48.22) |
| Female | 50.29 (49.89, 50.69) | 50.52 (50.14, 50.91) | 50.16 (49.57, 50.74) | 50.22 (49.74, 50.70) | 50.30 (50.06, 50.53) |
| Missing | 1.62 (1.17, 2.07) | 1.35 (0.89, 1.81) | 1.72 (0.92, 2.53) | 2.20 (1.46, 2.95) | 1.73 (1.41, 2.05) |
| **Age (years)** | | | | | |
| 18–34 | 21.14 (18.35, 23.92) | 23.11 (20.53, 25.70) | 22.90 (20.16, 25.65) | 25.47 (23.44, 27.50) | 23.17 (21.89, 24.44) |
| 35–49 | 27.67 (24.49, 30.85) | 26.09 (23.81, 28.37) | 24.60 (21.77, 27.43) | 24.80 (22.71, 26.89) | 25.78 (24.47, 27.09) |
| 50–64 | 29.03 (27.32, 30.75) | 29.79 (27.84, 31.75) | 30.07 (28.00, 32.14) | 26.95 (25.24, 28.66) | 28.95 (28.02, 29.89) |
| ≥ 65 | 18.63 (18.41, 18.85) | 18.99 (18.69, 19.30) | 19.53 (19.30, 19.76) | 19.98 (19.61, 20.35) | 19.29 (19.14, 19.43) |
| Missing | 3.53 (2.46, 4.60) | 2.02 (1.46, 2.57) | 2.90 (1.77, 4.02) | 2.80 (1.96, 3.64) | 2.81 (2.35, 3.27) |
| **Race/ethnicity** | | | | | |
| Non-Hispanic Asian | 5.10 (4.88, 5.32) | 4.76 (4.07, 5.45) | 4.93 (4.33, 5.53) | 4.83 (4.34, 5.32) | 4.90 (4.64, 5.17) |
| Non-Hispanic Black | 9.45 (8.58, 10.32) | 9.99 (9.49, 10.48) | 10.50 (10.02, 10.97) | 10.32 (9.76, 10.89) | 10.07 (9.76, 10.38) |
| Hispanic | 14.48 (14.01, 14.95) | 14.71 (14.16, 15.26) | 15.47 (15.18, 15.75) | 15.73 (15.55, 15.91) | 15.10 (14.90, 15.30) |
| Non-Hispanic White | 60.39 (59.39, 61.39) | 59.75 (58.76, 60.74) | 58.48 (57.54, 59.42) | 58.70 (57.58, 59.82) | 59.33 (58.82, 59.83) |
| Non-Hispanic Other** | 2.52 (2.33, 2.71) | 3.00 (2.51, 3.50) | 2.73 (2.40, 3.07) | 3.09 (2.61, 3.56) | 2.84 (2.64, 3.03) |
| Missing | 8.06 (6.64, 9.48) | 7.79 (6.39, 9.19) | 7.89 (6.51, 9.27) | 7.33 (5.98, 8.68) | 7.76 (7.07, 8.46) |
| **Sexual orientation** | | | | | |
| Heterosexual | 90.11 (88.42, 91.79) | 89.02 (86.97, 91.07) | 89.30 (87.69, 90.92) | 88.42 (86.90, 89.94) | 89.21 (88.34, 90.07) |

*H5C3 limited to paper-only responses and their corresponding sample weights.

**Includes American Indian, Alaska Native, Pacific Islander, Native Hawaiian, and multiracial adults.

| | H5C1 (*n* = 3,287) | H5C2 (*n* = 3,498) | H5C3* (*n* = 3,349) | H5C4 (*n* = 3,864) | Total (*N* = 13,993) |
|---|---|---|---|---|---|
| | **% (95% CI)** | **% (95% CI)** | **% (95% CI)** | **% (95% CI)** | **% (95% CI)** |
| **Sex** | | | | | |
| LGBTQ+ | 4.57 (2.92, 6.23) | 4.46 (2.84, 6.07) | 4.25 (2.87, 5.64) | 5.06 (3.76, 6.36) | 4.59 (3.84, 5.34) |
| Missing | 5.32 (4.39, 6.25) | 6.52 (4.99, 8.04) | 6.44 (5.08, 7.80) | 6.52 (5.44, 7.60) | 6.20 (5.58, 6.83) |
| **Annual household income** | | | | | |
| <$20,000 | 15.82 (14.00, 17.64) | 15.85 (13.55, 18.14) | 17.07 (13.98, 20.16) | 13.89 (12.21, 15.57) | 15.65 (14.50, 16.80) |
| $20,000 - $49,999 | 24.61 (22.47, 26.74) | 22.79 (20.44, 25.14) | 21.53 (19.13, 23.94) | 22.12 (20.15, 24.08) | 22.75 (21.65, 23.86) |
| $50,000 - $74,999 | 17.36 (15.43, 19.29) | 16.00 (13.83, 18.16) | 17.07 (14.96, 19.19) | 16.74 (14.03, 19.45) | 16.79 (15.66, 17.92) |
| ≥$75,000 | 32.99 (30.47, 35.52) | 35.29 (32.47, 38.12) | 35.27 (32.67, 37.87) | 38.92 (35.97, 41.88) | 35.64 (34.27, 37.00) |
| Missing | 9.22 (7.28, 11.16) | 10.07 (8.71, 11.44) | 9.06 (7.64, 10.48) | 8.33 (6.84, 9.82) | 9.17 (8.38, 9.95) |
| **Education** | | | | | |
| <High school | 8.47 (6.67, 10.27) | 8.86 (7.31, 10.41) | 7.14 (5.65, 8.63) | 7.81 (6.27, 9.36) | 8.07 (7.27, 8.87) |
| High school graduate | 22.48 (20.65, 24.32) | 21.97 (20.40, 23.53) | 23.12 (21.18, 25.06) | 21.89 (20.21, 23.58) | 22.36 (21.48, 23.24) |
| Some college, vocational, or technical training | 32.17 (30.62, 33.72) | 39.43 (37.76, 41.10) | 38.99 (37.11, 40.86) | 38.10 (36.42, 39.78) | 37.19 (36.34, 38.04) |
| College graduate or postgraduate | 34.85 (34.43, 35.26) | 28.38 (28.10, 28.65) | 28.76 (28.45, 29.06) | 29.44 (29.24, 29.64) | 30.34 (30.19, 30.49) |
| Missing | 2.03 (1.45, 2.61) | 1.37 (0.80, 1.94) | 2.00 (1.24, 2.75) | 2.76 (1.76, 3.75) | 2.04 (1.67, 2.42) |
| **Marital status** | | | | | |
| Single and never married | 29.32 (28.77, 29.86) | 29.95 (29.59, 30.31) | 29.45 (28.54, 30.35) | 29.86 (29.32, 30.41) | 29.64 (29.33, 29.96) |
| Married or living as married | 53.77 (52.92, 54.62) | 51.83 (50.52, 53.14) | 54.52 (53.47, 55.57) | 53.15 (52.01, 54.30) | 53.32 (52.77, 53.87) |
| Divorced, separated, or widowed | 14.27 (13.58, 14.96) | 16.89 (15.56, 18.22) | 13.69 (12.80, 14.58) | 13.99 (13.25, 14.74) | 14.71 (14.23, 15.18) |
| Missing | 2.64 (1.81, 3.47) | 1.33 (0.85, 1.82) | 2.34 (1.26, 3.41) | 2.99 (1.99, 4.00) | 2.33 (1.89, 2.77) |

*H5C3 limited to paper-only responses and their corresponding sample weights.

**Includes American Indian, Alaska Native, Pacific Islander, Native Hawaiian, and multiracial adults.

| | H5C1 (*n* = 3,287) | H5C2 (*n* = 3,498) | H5C3[*] (*n* = 3,349) | H5C4 (*n* = 3,864) | Total (*N* = 13,993) |
|---|---|---|---|---|---|
| | % (95% CI) | % (95% CI) | % (95% CI) | % (95% CI) | % (95% CI) |
| **Sex** | | | | | |
| **Adults in household** | | | | | |
| 1 | 18.20 (16.06, 20.34) | 19.01 (17.17, 20.85) | 20.76 (18.68, 22.83) | 18.00 (16.35, 19.65) | 18.99 (18.03, 19.96) |
| ≥ 2 | 81.80 (79.66, 83.94) | 80.99 (79.15, 82.83) | 79.24 (77.17, 81.32) | 82.00 (80.35, 83.65) | 81.01 (80.04, 81.97) |
| **Children in household** | | | | | |
| 0 | 62.84 (60.22, 65.46) | 65.63 (63.39, 67.87) | 63.78 (61.10, 66.47) | 61.83 (59.36, 64.30) | 63.51 (62.26, 64.77) |
| ≥ 1 | 30.74 (28.22, 33.27) | 28.35 (26.40, 30.30) | 29.43 (27.18, 31.68) | 32.15 (29.28, 35.03) | 30.18 (28.96, 31.39) |
| Missing | 6.42 (5.09, 7.74) | 6.02 (4.50, 7.54) | 6.79 (5.04, 8.53) | 6.01 (4.55, 7.48) | 6.31 (5.55, 7.07) |
| **Rural/urban residency** | | | | | |
| Metropolitan | 85.82 (84.02, 87.62) | 86.30 (84.54, 88.06) | 86.77 (84.95, 88.59) | 87.76 (86.29, 89.23) | 86.67 (85.81, 87.53) |
| Non-metro urban | 12.26 (10.62, 13.89) | 12.65 (10.84, 14.45) | 11.89 (10.03, 13.74) | 11.20 (9.76, 12.64) | 11.99 (11.15, 12.84) |
| Non-metro rural | 1.93 (1.12, 2.74) | 1.06 (0.62, 1.49) | 1.34 (0.79, 1.90) | 1.04 (0.62, 1.45) | 1.34 (1.05, 1.63) |
| **Census region** | | | | | |
| Northeast | 17.90 (17.85, 17.94) | 17.81 (17.79, 17.84) | 17.69 (17.55, 17.82) | 17.54 (17.54, 17.54) | 17.73 (17.70, 17.77) |
| Midwest | 21.11 (21.09, 21.13) | 20.97 (20.91, 21.04) | 20.94 (20.87, 21.00) | 20.83 (20.83, 20.84) | 20.96 (20.94, 20.98) |
| South | 37.54 (37.50, 37.57) | 37.62 (37.54, 37.69) | 37.73 (37.66, 37.80) | 37.92 (37.91, 37.92) | 37.70 (37.68, 37.73) |
| West | 23.46 (23.40, 23.52) | 23.60 (23.56, 23.63) | 23.65 (23.58, 23.72) | 23.71 (23.70, 23.71) | 23.60 (23.58, 23.63) |
| **Health insurance coverage** | | | | | |
| Yes | 90.62 (90.13, 91.10) | 89.89 (89.28, 90.51) | 90.08 (89.19, 90.97) | 89.78 (89.13, 90.44) | 90.09 (89.75, 90.43) |
| No | 8.17 (8.14, 8.19) | 8.33 (8.21, 8.44) | 8.27 (8.18, 8.37) | 8.88 (8.73, 9.03) | 8.41 (8.36, 8.47) |
| Missing | 1.22 (0.74, 1.70) | 1.78 (1.18, 2.38) | 1.65 (0.74, 2.56) | 1.34 (0.68, 1.99) | 1.49 (1.15, 1.84) |

[*] H5C3 limited to paper-only responses and their corresponding sample weights.

[**] Includes American Indian, Alaska Native, Pacific Islander, Native Hawaiian, and multiracial adults.

| | H5C1 (*n* = 3,287) | H5C2 (*n* = 3,498) | H5C3* (*n* = 3,349) | H5C4 (*n* = 3,864) | Total (*N* = 13,993) |
|---|---|---|---|---|---|
| | **% (95% CI)** | **% (95% CI)** | **% (95% CI)** | **% (95% CI)** | **% (95% CI)** |
| **Sex** | | | | | |
| **Regular healthcare provider** | | | | | |
| Yes | 64.67 (62.09, 67.24) | 64.47 (61.90, 67.04) | 62.83 (59.68, 65.98) | 61.39 (58.97, 63.81) | 63.33 (61.98, 64.68) |
| No | 34.29 (31.67, 36.91) | 33.86 (31.29, 36.43) | 35.02 (31.83, 38.22) | 37.22 (34.89, 39.55) | 35.11 (33.76, 36.46) |
| Missing | 1.05 (0.60, 1.50) | 1.67 (0.76, 2.58) | 2.15 (1.07, 3.22) | 1.38 (0.94, 1.83) | 1.56 (1.18, 1.95) |
| **Healthcare visit in past year** | | | | | |
| Yes | 81.75 (79.49, 84.01) | 79.98 (77.32, 82.64) | 82.57 (80.03, 85.12) | 82.65 (80.94, 84.35) | 81.74 (80.58, 82.90) |
| No | 17.00 (14.77, 19.22) | 18.93 (16.42, 21.44) | 16.62 (14.06, 19.18) | 16.74 (14.98, 18.50) | 17.32 (16.18, 18.46) |
| Missing | 1.26 (0.80, 1.72) | 1.09 (0.29, 1.89) | 0.81 (0.22, 1.40) | 0.61 (0.37, 0.86) | 0.94 (0.66, 1.22) |
| **General health** | | | | | |
| Excellent, very good, good | 82.23 (79.79, 84.68) | 84.69 (82.84, 86.54) | 83.36 (80.94, 85.77) | 85.28 (83.54, 87.02) | 83.90 (82.83, 84.96) |
| Fair or poor | 16.86 (14.50, 19.21) | 14.71 (12.84, 16.58) | 15.05 (12.81, 17.30) | 14.01 (12.29, 15.72) | 15.15 (14.12, 16.18) |
| Missing | 0.91 (0.47, 1.36) | 0.60 (0.35, 0.85) | 1.59 (0.71, 2.47) | 0.71 (0.37, 1.06) | 0.96 (0.69, 1.22) |
| **Chronic health conditions** | | | | | |
| 0 | 47.53 (44.94, 50.12) | 48.71 (46.24, 51.19) | 48.66 (45.95, 51.38) | 46.51 (44.61, 48.41) | 47.85 (46.63, 49.07) |
| 1 | 28.05 (25.47, 30.62) | 27.14 (24.91, 29.38) | 27.23 (24.70, 29.75) | 28.76 (26.48, 31.05) | 27.80 (26.59, 29.00) |
| ≥ 2 | 21.23 (19.51, 22.94) | 20.83 (19.09, 22.58) | 20.51 (18.50, 22.51) | 21.47 (19.65, 23.30) | 21.01 (20.10, 21.93) |
| Missing | 3.20 (2.35, 4.05) | 3.31 (2.39, 4.23) | 3.60 (2.63, 4.58) | 3.25 (2.40, 4.10) | 3.34 (2.89, 3.79) |
| **Depression or anxiety disorder** | | | | | |
| Yes | 22.74 (20.32, 25.16) | 23.69 (21.08, 26.30) | 22.85 (20.23, 25.46) | 24.07 (21.97, 26.18) | 23.34 (22.12, 24.56) |
| No | 75.50 (73.10, 77.90) | 74.23 (71.43, 77.02) | 75.15 (72.42, 77.88) | 74.93 (72.79, 77.08) | 74.95 (73.69, 76.22) |

*H5C3 limited to paper-only responses and their corresponding sample weights.

**Includes American Indian, Alaska Native, Pacific Islander, Native Hawaiian, and multiracial adults.

| | H5C1 (*n* = 3,287) | H5C2 (*n* = 3,498) | H5C3* (*n* = 3,349) | H5C4 (*n* = 3,864) | Total (*N* = 13,993) |
|---|---|---|---|---|---|
| | % (95% CI) | % (95% CI) | % (95% CI) | % (95% CI) | % (95% CI) |
| **Sex** | | | | | |
| Missing | 1.76 (1.08, 2.44) | 2.08 (1.29, 2.88) | 2.00 (1.32, 2.68) | 0.99 (0.58, 1.40) | 1.71 (1.38, 2.03) |
| **Weekly physical activity (minutes)** | | | | | |
| < 150 | 57.54 (54.61, 60.47) | 64.20 (61.55, 66.84) | 62.01 (58.98, 65.03) | 59.30 (56.25, 62.36) | 60.76 (59.30, 62.22) |
| ≥ 150 | 41.11 (38.28, 43.94) | 32.81 (30.26, 35.36) | 34.53 (31.84, 37.22) | 37.24 (34.47, 40.00) | 36.41 (35.06, 37.77) |
| Missing | 1.35 (0.92, 1.79) | 2.99 (2.23, 3.76) | 3.46 (2.34, 4.59) | 3.46 (2.12, 4.81) | 2.83 (2.33, 3.32) |
| *H5C3 limited to paper-only responses and their corresponding sample weights. | | | | | |
| **Includes American Indian, Alaska Native, Pacific Islander, Native Hawaiian, and multiracial adults. | | | | | |

**Digital technologies access and health use patterns (Aim 1).** A ten-class model (Fig. 1) of distinct digital technology access and health use patterns emerged from Step 1 of the LCA with a balance of high entropy and good fit (Table 2). Classes 1 and 2 included digitally isolated and mobile-dependent individuals who made up an estimated 8.9% and 7.8% of US adults, respectively. Health uses of digital technologies among members of class 1 included texting healthcare providers (7.8%), tracking their health with wearables (1.8%), and sharing data from monitoring devices with healthcare providers (8.5%) (Fig. 1). Roughly 25% of class 2 members engaged in mobile-based health behaviors, ranging from 5.3% who used wearable devices to track their health to 23.8% who used smart mobile devices to make medical treatment decisions.

Table 2
Model fit information and selection criteria for latent class models with 1 to 20 classes

| Classes | # Parameters | Log-likelihood | Entropy | AIC | CAIC | BIC | SABIC | VLMR-LR |
|---|---|---|---|---|---|---|---|---|
| Desideratum: | - | - | > 0.8 | Lowest value | Lowest value | Lowest value | Lowest value | Greater magnitude indicates greater improvement over the previous model* |
| 1 | 64 | -299539 | - | 599206 | 599753 | 599689 | 599485 | - |
| 2 | 129 | -214242 | 0.99 | 428742 | 429845 | 429716 | 429306 | 164838 |
| 3 | 194 | -172165 | 0.99 | 344718 | 346376 | 346182 | 345566 | 81315 |
| 4 | 259 | -159902 | 0.96 | 320321 | 322535 | 322276 | 321453 | 23700 |
| 5 | 324 | -154752 | 0.96 | 310152 | 312921 | 312597 | 311567 | 9952 |
| 6 | 389 | -149695 | 0.97 | 300168 | 303493 | 303104 | 301868 | 9772 |
| 7 | 454 | -146777 | 0.97 | 294461 | 298341 | 297887 | 296445 | 5640 |
| 8 | 519 | -143929 | 0.94 | 288896 | 293332 | 292813 | 291163 | 5503 |
| 9 | 584 | -141397 | 0.93 | 283962 | 288953 | 288369 | 286513 | 4893 |
| **10** | **649** | **-139854** | **0.92** | **281007** | **286553** | **285904** | **283842** | **2981** |
| 11 | 714 | -139262 | 0.91 | 279952 | 286054 | 285340 | 283071 | 1145 |
| 12 | 779 | -138713 | 0.90 | 278984 | 285641 | 284862 | 282387 | 1061 |
| 13 | 844 | -138198 | 0.89 | 278085 | 285298 | 284454 | 281772 | 994 |
| 14 | 909 | -137722 | 0.88 | 277262 | 285031 | 284122 | 281233 | 920 |
| 15 | 974 | -137292 | 0.88 | 276533 | 284857 | 283883 | 280788 | 831 |
| 16 | 1039 | -136895 | 0.88 | 275867 | 284747 | 283708 | 280406 | 769 |
| 17 | 1104 | -136561 | 0.88 | 275330 | 284765 | 283661 | 280152 | 645 |
| 18 | 1169 | -136233 | 0.88 | 274805 | 284796 | 283627 | 279912 | 633 |
| 19 | 1234 | -135978 | 0.87 | 274423 | 284969 | 283735 | 279814 | 494 |
| 20 | 1299 | -135749 | 0.87 | 274096 | 285198 | 283899 | 279771 | 442 |

Fit for latent class models without covariates.

Bold indicates selected model.

AIC = Akaike information criterion, BIC = Bayesian information criterion, CAIC = Consistent AIC, SABIC = Sample size adjusted BIC, VLMR-LR = Vuong-Lo-Mendell-Rubin adjusted likelihood ratio (of *k* class model to *k*-1 class model, hence none for 1 class model).

*All likelihood ratio tests have $p < 0.001$, indicating statistically significant improvement of each k-class model compared to previous k-1 class model.

Members of classes 3 and 4, which made up 2.3% and 2.1% of US adults, lacked access to mobile devices but were digitally connected via internet-enabled computers/laptops and basic cell phones. The primary behavior that members of classes 3 and 4 engaged in was online health information seeking for oneself (49.9%, 65.3%) and for someone else (32.6%, 39.9%). Roughly < 20% and < 40% of class 3 and 4 members engaged in all other web-based behaviors. Unlike class 3, class 4 members had access to EHRs with an estimated 43.5% of its members logging in to their own EHRs. Outside of viewing test results, use of EHR features was low with < 10% of class members using 6 features and between 10% and 20% of members using 3 features.

Class 5 and 6 members shared access to internet-enabled devices but lacked access to EHRs. These classes made up 17.2% and 13.0% of US adults, respectively. Except for online health information seeking, roughly ≤ 30% of class 5 members and ≤ 75% of class 6 members engaged in web- and mobile device-based health behaviors. These behaviors ranged from participating in online health forums or support groups (1.3% and 13.1%) to seeking healthcare provider online (28.1% and 73.7%) among class 5 and 6 members. Noteworthy, class 6 had the second highest percentage of using smart mobile devices (66.8%) and wearables (30.7%) to track health and health goals and sharing of tracked data with healthcare providers (21.1%).

Classes 7 through 10 had complete access to internet-enabled devices and EHRs. Combined, members of these classes made up 48.7% of US adults. One notable difference among them is the sparse use of EHRs among members of classes 7 and 8 where only 12% logged in to their online EHR in the past year and use of any EHR features was almost nonexistent. Members of other classes with EHR access had notably more utilization: 43.5% of class 4 members and 100% of classes 9 and 10 members had logged in to their own EHR. Of 10 EHR features examined, viewing test results and communicating with healthcare providers were the most used across classes 4, 9, and 10. Additionally, > 25% of class 9 members used 5 EHR features and > 50% of class 10 members used 6 EHR features.

Classes 7 through 10 also differed in the percentage of their members who engaged in health uses of digital technologies. Class 7 members exhibited low use;<30% of its members engaged in any behavior (except online health information seeking). Classes 8 and 9 members exhibited moderate-to-high use of most digital health technologies. A higher percentage of class 8 members used mobile technologies than class 9 members (e.g., 57.8% of class 8 members used smart mobile devices to make medical treatment decisions vs. 32.9% of class 9). However, the two classes were nearly identical in other web-based health behaviors (e.g., 49.0% of class 8 members tracked healthcare costs online vs. 49.6% of class 9). Making up 13.6% of US adults, class 10 consisted of super users of all 32 health behaviors examined. Between 50% and 80% of class members engaged in 12 behaviors and > 80% engaged in 10 behaviors. Furthermore, class 10 members engaged in uncommon or nonexistent behaviors among members of other classes (e.g., sharing health information on social networking sites (35.3%), health tracking using wearables (40.0%)).

**Associations between sociodemographic characteristics, health factors, and class membership (Aim 2).** Age and education were associated with class membership (Table 3). For example, adults aged 50–64 had 19.8 times the odds and those aged 65 + had 199.1 times the odds of class 1 (vs. class 10) membership compared to those aged 18–34. Adults with less than high school education had 690.9 times the odds, those with high school education had 29.2 times the odds, and those with some college, vocational, or technical training had 5.7 times the odds of class 1 (vs. class 10) membership compared to college graduates and those with postgraduate degrees. Sex was also associated with class membership where females had lower odds of belonging to classes 1 through 6 (vs. class 10) than males (aORs ranged from 0.29 for class 3 to 0.56 for class 2). Non-Hispanic Asian (aOR = 2.11) and Hispanic (aOR = 2.68) adults had greater odds of belonging to class 2 (vs. class 10) than non-Hispanic White adults. Others correlates of class membership included marital status and rural/urban residency, whereas sexual orientation, census regions, and the numbers of adults and children in the household were largely not associated with class membership.

Table 3
Multinomial logistic regression of sociodemographics and health factors onto class membership, ($n$ = 10,960, class 10 is reference class)

| **Covariate** | **Class 1** aOR (95% CI) | **Class 2** aOR (95% CI) | **Class 3** aOR (95% CI) | **Class 4** aOR (95% CI) | **Class 5** aOR (95% CI) | **Class 6** aOR (95% CI) | **Class 7** aOR (95% CI) | **Class 8** aOR (95% CI) | **Class 9** aOR (95% CI) |
|---|---|---|---|---|---|---|---|---|---|
| **Sex** (ref: Male) | | | | | | | | | |
| Female | **0.34 [0.24, 0.49]** | **0.56 [0.38, 0.84]** | **0.29 [0.15, 0.54]** | **0.47 [0.29, 0.76]** | **0.36 [0.26, 0.52]** | **0.54 [0.38, 0.76]** | 0.55 [0.29, 1.03] | 0.88 [0.48, 1.62] | 0.80 [0.53, 1.19] |
| **Age** (ref: 18–34) | | | | | | | | | |
| 35–49 | 3.27 [0.85, 12.53] | **2.39 [1.06, 5.42]** | 4.81 [0.80, 29.00] | 3.18 [0.59, 17.09] | 1.24 [0.68, 2.30] | 0.72 [0.40, 1.31] | 1.20 [0.22, 6.49] | 0.89 [0.26, 3.05] | 1.43 [0.66, 3.09] |
| 50–64 | **19.81 [5.50, 71.29]** | **6.23 [2.82, 13.77]** | **32.14 [6.24, 165.59]** | **6.71 [1.76, 25.61]** | **2.28 [1.34, 3.90]** | 1.26 [0.69, 2.32] | **3.11 [1.04, 9.28]** | 1.22 [0.43, 3.44] | **2.91 [1.52, 5.57]** |
| ≥ 65 | **199.14 [55.94, 708.96]** | **20.68 [9.31, 45.92]** | **114.21 [21.47, 607.36]** | **43.90 [10.71, 179.97]** | **4.47 [2.49, 8.02]** | 0.90 [0.40, 2.06] | **6.29 [2.06, 19.25]** | 0.98 [0.47, 2.06] | **4.06 [2.03, 8.10]** |
| **Race/ethnicity** (ref: NHW) | | | | | | | | | |
| NHA | 1.46 [0.41, 5.20] | **2.11 [1.03, 4.30]** | 0.42 [0.06, 2.75] | 0.12 [< 0.01, 11.11] | 0.76 [0.34, 1.69] | 0.99 [0.48, 2.04] | 0.41 [0.12, 1.36] | 0.47 [0.19, 1.17] | 0.84 [0.42, 1.66] |
| NHB | 1.04 [0.57, 1.90] | 1.53 [0.84, 2.77] | 0.78 [0.22, 2.72] | 0.60 [0.12, 2.99] | 0.67 [0.27, 1.70] | 1.27 [0.61, 2.66] | 0.57 [0.18, 1.79] | 1.14 [0.38, 3.40] | **0.52 [0.28, 0.93]** |
| Hispanic | 1.56 [0.83, 2.94] | **2.68 [1.63, 4.41]** | 0.51 [0.23, 1.15] | 1.18 [0.58, 2.39] | 1.08 [0.68, 1.72] | 1.43 [0.89, 2.29] | 0.78 [0.33, 1.82] | 1.23 [0.48, 3.10] | 0.66 [0.38, 1.14] |
| NHO | 0.93 [0.24, 3.70] | 0.67 [0.18, 2.52] | 0.17 [0.01, 2.02] | 0.73 [0.13, 4.23] | 0.95 [0.30, 3.00] | 0.72 [0.23, 2.23] | 0.27 [0.03, 2.70] | 0.92 [0.25, 3.45] | 0.66 [0.23, 1.91] |
| **Sexual orientation** (ref: Heterosexual) | | | | | | | | | |
| Non-heterosexual | 0.45 [0.14, 1.45] | **0.21 [0.05, 0.86]** | **0.12 [0.02, 0.63]** | 0.84 [0.26, 2.73] | 0.40 [0.16, 1.02] | 0.51 [0.24, 1.07] | **0.16 [0.03, 0.84]** | 0.48 [0.18, 1.27] | 0.65 [0.28, 1.49] |
| **Education** (ref: College graduate or postgraduate) | | | | | | | | | |

aOR = adjusted odds ratio, CI = confidence intervals.

Bold adjusted odds ratios and confidence intervals do not include 1.

| Covariate | Class 1 aOR (95% CI) | Class 2 aOR (95% CI) | Class 3 aOR (95% CI) | Class 4 aOR (95% CI) | Class 5 aOR (95% CI) | Class 6 aOR (95% CI) | Class 7 aOR (95% CI) | Class 8 aOR (95% CI) | Class 9 aOR (95% CI) |
|---|---|---|---|---|---|---|---|---|---|
| <High school | **690.90 [50.24, 9501.58]** | **118.27 [8.17, 1713.04]** | **138.93 [8.19, 2356.88]** | **48.38 [2.65, 884.08]** | **39.85 [3.20, 495.77]** | 7.39 [0.50, 110.12] | **45.70 [2.84, 735.57]** | 3.21 [0.16, 63.43] | 9.07 [0.53, 156.18] |
| High school graduate | **29.25 [16.08, 53.21]** | **10.51 [6.23, 17.73]** | **7.08 [3.70, 13.54]** | **4.57 [2.30, 9.06]** | **4.49 [2.74, 7.36]** | 1.52 [0.87, 2.67] | **4.91 [1.92, 12.56]** | 1.24 [0.34, 4.47] | 1.25 [0.72, 2.14] |
| Some college, vocational, or technical training | **5.73 [3.30, 9.95]** | **3.13 [2.01, 4.88]** | **3.60 [2.15, 6.02]** | **1.99 [1.02, 3.88]** | **2.95 [1.92, 4.52]** | **1.52 [1.00, 2.29]** | **2.80 [1.07, 7.35]** | 1.56 [0.91, 2.68] | 1.41 [0.86, 2.34] |
| **Marital status** (ref: Single and never married) | | | | | | | | | |
| Married or living with partner | **0.24 [0.11, 0.52]** | 0.52 [0.25, 1.08] | **0.16 [0.07, 0.38]** | **0.25 [0.10, 0.61]** | **0.51 [0.26, 0.97]** | 0.75 [0.40, 1.40] | 0.47 [0.15, 1.47] | 1.34 [0.39, 4.65] | 1.11 [0.39, 3.14] |
| Divorced, separated, or widowed | 0.81 [0.44, 1.50] | 1.00 [0.54, 1.86] | 0.64 [0.24, 1.66] | **0.45 [0.24, 0.87]** | 1.10 [0.61, 2.01] | 1.00 [0.53, 1.90] | 0.93 [0.27, 3.23] | 1.46 [0.34, 6.16] | 1.17 [0.53, 2.57] |
| **Adults in household** (ref: 1) | | | | | | | | | |
| ≥ 2 | **0.48 [0.24, 0.95]** | 0.53 [0.26, 1.08] | 0.58 [0.26, 1.27] | 0.70 [0.30, 1.64] | 0.76 [0.41, 1.40] | 1.00 [0.52, 1.92] | 1.19 [0.52, 2.71] | 0.68 [0.32, 1.44] | 0.67 [0.29, 1.55] |
| **Children in household** (ref: 0) | | | | | | | | | |
| ≥ 1 | 0.62 [0.34, 1.13] | 0.90 [0.55, 1.49] | 0.61 [0.27, 1.40] | **0.29 [0.10, 0.89]** | 0.90 [0.61, 1.34] | 1.10 [0.78, 1.56] | 0.93 [0.56, 1.56] | 1.05 [0.64, 1.72] | 0.98 [0.71, 1.36] |
| **Rural/urban residency** (ref: Metropolitan) | | | | | | | | | |
| Non-metro urban | **2.53 [1.43, 4.46]** | **1.97 [1.12, 3.47]** | 1.32 [0.61, 2.86] | 1.37 [0.72, 2.61] | **2.21 [1.29, 3.76]** | 1.44 [0.79, 2.62] | **2.13 [1.12, 4.07]** | 1.32 [0.56, 3.11] | 1.28 [0.75, 2.18] |
| Non-metro rural | **4.72 [1.26, 17.70]** | 1.14 [0.26, 4.95] | 2.42 [0.59, 9.91] | 1.48 [0.28, 7.83] | 1.61 [0.55, 4.77] | 1.82 [0.51, 6.47] | 1.06 [0.31, 3.64] | 1.26 [0.32, 4.98] | 0.68 [0.21, 2.24] |
| **Census region** (ref: Northeast) | | | | | | | | | |
| Midwest | 0.85 [0.45, 1.61] | 0.99 [0.55, 1.78] | 0.88 [0.40, 1.93] | 0.75 [0.32, 1.76] | 0.82 [0.49, 1.38] | 0.61 [0.36, 1.03] | 0.77 [0.37, 1.59] | 0.61 [0.24, 1.51] | 0.80 [0.49, 1.32] |

aOR = adjusted odds ratio, CI = confidence intervals.

Bold adjusted odds ratios and confidence intervals do not include 1.

| Covariate | Class 1 aOR (95% CI) | Class 2 aOR (95% CI) | Class 3 aOR (95% CI) | Class 4 aOR (95% CI) | Class 5 aOR (95% CI) | Class 6 aOR (95% CI) | Class 7 aOR (95% CI) | Class 8 aOR (95% CI) | Class 9 aOR (95% CI) |
|---|---|---|---|---|---|---|---|---|---|
| South | 0.69 [0.39, 1.23] | 0.87 [0.51, 1.47] | 0.57 [0.29, 1.13] | 0.69 [0.31, 1.56] | 0.72 [0.40, 1.31] | 1.06 [0.65, 1.75] | 0.65 [0.39, 1.09] | 0.89 [0.46, 1.70] | 0.91 [0.51, 1.62] |
| West | **0.48 [0.26, 0.86]** | 0.66 [0.39, 1.11] | **0.38 [0.18, 0.83]** | 0.86 [0.40, 1.81] | **0.53 [0.31, 0.91]** | 0.86 [0.53, 1.39] | **0.44 [0.24, 0.80]** | 0.88 [0.41, 1.91] | 0.76 [0.46, 1.25] |
| **Health insurance coverage** (ref: Yes) | | | | | | | | | |
| No | 2.06 [0.60, 7.11] | 2.56 [0.83, 7.95] | 2.93 [0.54, 15.98] | 2.40 [0.34, 16.71] | 1.93 [0.61, 6.16] | 1.91 [0.52, 7.05] | 1.32 [0.12, 14.04] | 1.41 [0.23, 8.59] | 0.74 [0.16, 3.38] |
| **Regular healthcare provider** (ref: Yes) | | | | | | | | | |
| No | **2.69 [1.54, 4.71]** | **3.23 [1.85, 5.63]** | **2.93 [1.41, 6.09]** | 1.36 [0.59, 3.17] | **4.54 [2.87, 7.18]** | **2.84 [1.71, 4.72]** | **2.47 [1.32, 4.62]** | **2.73 [1.59, 4.67]** | 1.57 [0.85, 2.92] |
| **Healthcare visit in past year** (ref: Yes) | | | | | | | | | |
| No | **6.65 [3.10, 14.23]** | **4.20 [2.05, 8.57]** | **4.68 [1.68, 13.09]** | 1.25 [0.35, 4.44] | **4.83 [2.49, 9.36]** | 2.05 [0.95, 4.43] | 2.23 [0.51, 9.68] | 2.42 [0.65, 9.01] | 1.13 [0.47, 2.76] |
| **General health** (ref: Excellent, very good, or good) | | | | | | | | | |
| Fair or Poor | **2.90 [1.72, 4.91]** | 1.42 [0.84, 2.41] | 1.95 [0.87, 4.40] | 2.08 [0.93, 4.65] | 1.29 [0.78, 2.12] | 1.17 [0.72, 1.88] | 1.13 [0.40, 3.18] | 1.29 [0.52, 3.20] | 1.01 [0.60, 1.70] |
| **Chronic health conditions** (ref: 0) | | | | | | | | | |
| 1 | **0.44 [0.25, 0.77]** | **0.51 [0.30, 0.86]** | **0.46 [0.23, 0.90]** | 0.50 [0.23, 1.08] | **0.46 [0.27, 0.77]** | **0.50 [0.32, 0.79]** | **0.49 [0.25, 0.95]** | 0.63 [0.34, 1.15] | 0.60 [0.35, 1.02] |
| ≥ 2 | **0.39 [0.23, 0.67]** | **0.43 [0.25, 0.73]** | **0.25 [0.12, 0.49]** | **0.48 [0.24, 0.97]** | **0.31 [0.18, 0.54]** | **0.42 [0.26, 0.69]** | **0.40 [0.24, 0.68]** | **0.42 [0.21, 0.84]** | **0.45 [0.28, 0.74]** |
| **Depression or anxiety disorder** (ref: No) | | | | | | | | | |

aOR = adjusted odds ratio, CI = confidence intervals.

Bold adjusted odds ratios and confidence intervals do not include 1.

| Covariate | Class 1<br><br>aOR (95% CI) | Class 2<br><br>aOR (95% CI) | Class 3<br><br>aOR (95% CI) | Class 4<br><br>aOR (95% CI) | Class 5<br><br>aOR (95% CI) | Class 6<br><br>aOR (95% CI) | Class 7<br><br>aOR (95% CI) | Class 8<br><br>aOR (95% CI) | Class 9<br><br>aOR (95% CI) |
|---|---|---|---|---|---|---|---|---|---|
| Yes | **0.35 [0.20, 0.60]** | 0.57 [0.32, 1.03] | 0.94 [0.44, 2.02] | **0.41 [0.22, 0.79]** | **0.46 [0.29, 0.74]** | 0.78 [0.52, 1.16] | 0.57 [0.31, 1.06] | 0.74 [0.40, 1.35] | 0.61 [0.36, 1.05] |
| **Weekly physical activity** (ref: <150 minutes) | | | | | | | | | |
| ≥ 150 minutes (meets HHS recommendation) | **0.50 [0.32, 0.78]** | **0.55 [0.36, 0.85]** | 0.75 [0.45, 1.26] | **0.42 [0.26, 0.70]** | **0.54 [0.35, 0.85]** | 0.80 [0.53, 1.22] | 0.67 [0.31, 1.45] | 0.84 [0.37, 1.87] | 0.64 [0.41, 1.00] |
| aOR = adjusted odds ratio, CI = confidence intervals. | | | | | | | | | |
| Bold adjusted odds ratios and confidence intervals do not include 1. | | | | | | | | | |

Adults who reported not having a regular healthcare provider or not visiting a provider in the past year had greater odds of membership in classes 1 through 3 or 5 (vs. class 10) than adults who reported having a regular provider or having visited one in the past year. For example, adults who reported not having a regular health care provider (aOR = 2.69) and not visiting one in the past year (aOR = 6.65) had greater odds of belonging to class 1 (vs. class 10) than those reporting having or visiting a healthcare provider. The presence of chronic diseases was also associated with class membership. Adults with ≥2 chronic diseases had lower odds of belonging to all classes (vs. class 10) than those with no chronic conditions (aORs ranged from 0.25 for class 3 to 0.48 for class 4). Additionally, adults who reported exercising 150 + minutes/week had roughly half the odds of belonging to classes 1, 2, 4, or 5 (vs. class 10) than those who reported exercising < 150 minutes/week. Health insurance status and self-reported general health were largely not associated with class membership.

## Discussion

We identified ten unique digital technology access and health use patterns among a nationally representative sample of US adults. Roughly 50% of US adults had universal access to the internet and internet-enabled devices, smart mobile devices, and to their EHRs. The remaining half of US adults belonged to classes that lacked access to 1 + of these digital technologies. Within classes, the estimated proportions of members engaging in various health behaviors ranged from small to large. Disparate access to and health use of digital technologies was observed primarily by birth sex, age, educational attainment, and health factors. Specifically, digital technologies access and health use were lower among male, less educated, and older adults, while the relationship between race/ethnicity and access and use was weaker by comparison. The health factors most associated with membership of classes with lesser digital technologies access and health use were not having a regular healthcare provider, not visiting a provider in the past year, and not having any chronic diseases. These results have important implications. From health education to chronic disease management and behavior change, benefits of digital technologies use on health outcomes are well documented.[4,8,11,12,20] Identifying groups with common digital technologies access and health use patterns is critical for efforts aimed at improving access to digital technologies and increasing their health use among US adults to maximize individual- and population-level health benefits.

Our results make evident the lack of access to digital technologies among US adults. First, ~ 50% of US adults lacked EHR access (classes 1 through 3, 5, and 6) despite an accelerated rate of EHR adoption attributed, in part, to policies that incentivized adoption and meaningful use of EHRs.[52] Second, ~ 13% lacked access to both smartphones and tablets

(classes 1, 3, and 4), which aligns with national data on smartphone adoption rates.[23] Wearable device access was highest among class 10 members (52.5%), whereas wearables access was < 13% among members of six classes that made up half of US adults (classes 1 through 5 and 7). Third, ~ 16% of US adults did not utilize the internet (classes 1 and 2), despite class 2 members having access to internet-capable devices (e.g., smartphones, tablets). By default, health uses of digital technologies were nonexistent in classes missing access to requisite technologies, eliminating any possible benefits associated with their use. Thus, it is essential to monitor national targets (e.g., HealthyPeople 2030) for increasing access to digital technologies[53] and expand access to underserved populations through programs such as phone and internet service payment assistance and alternative third-party personal health record apps.[54,55]

Digital technologies access and health use patterns are constantly changing. Future studies should replicate the current work to examine the evolution in the classes identified here over time. For example, the digitally isolated class 1 could disappear if trends in adoption of digital technologies continue or as the aging members of this class die out. Potential future scenarios include the emergence of classes that reflect disparate access to newer technologies (e.g., smart home assistants) as other technologies (e.g., wearables) become mainstream.[56] Future research should also document access-driven disparities in health outcomes among adults who belong to classes with no/limited access to digital technologies and whether such disparities vary by individual histories of access (e.g., duration with uninterrupted access rather than by estimates of access at a single time point). Studies should also examine whether there are advantages to having access to multiple technologies that ostensibly facilitate the same health behavior. For example, given that mobile texting, email, and patient portals may all be used for patient-provider communication, does communication frequency and associated health outcome (e.g., patient satisfaction) differ depending on the type or number of technologies available to the patient?

Across classes, percentages of US adults using digital technologies for health varied. Consistent with previously published literature, seeking health information online was common across all web-integrated classes, and – of the queried EHR features – adults commonly viewed test results and communicated with their healthcare providers.[2,32,57] On digital technologies health use, several observations are noteworthy. First, while health uses of digital technologies are associated with positive health outcomes, evidence of positive outcomes is not definitive and unintended outcomes exist. For example, online health information seeking has been associated with unintended, often negative, outcomes (e.g., health misinformation).[58] Similarly, benefits of patient portals use on clinical health outcomes is inconclusive.[59,60] This introduces complexity in determining which technologies are potentially beneficial to health and the desired proportions of US adults engaging with digital health technologies, which potentially explains why national initiatives set goals solely for increasing access to digital technologies.[53]

Our results suggest the need to disentangle lack of access from nonuse, as the lines between them are often blurred. For example, limited use of EHRs can be attributed to a lack of access among people without health insurance or a regular healthcare provider (rather than an unwillingness to adopt them). Alternatively, EHR nonuse can be attributed to lack of (perceived) need, lack of awareness, and poor usability, among other factors.[61] Identifying factors associated with nonuse is critical to employing appropriate approaches to intervene on modifiable factors to reduce digital health disparities. Interventions should also target various interdependent factors commonly associated with use of digital technologies including individual predispositions (e.g., mistrust, privacy concerns), skills (e.g., limited digital literacy), and technology-related factors (e.g., poor usability).[28,29,62–64] Finally, although our analysis was limited to binary measures of health behaviors, frequency and duration of use can vary. Thus, it is important to consider how health outcomes may relate to the frequency of health behaviors and identify classes of adults based on levels of health use within and across digital technologies and health outcomes among adults who belong to these classes.

Our results feature a subset of US adults who use digital technologies in relative isolation from the traditional healthcare system, whether by choice (i.e., classes 7 and 8) or because they lacked access to their EHRs (i.e., classes 1 through 3, 5, and 6). Members of these classes utilized general web-based tools serving the same purpose as EHR features (e.g., communicating with provider, requesting medication refills), which illustrates the utility of these tools outside of patient portals and may explain the lag in EHR use among those with EHR access. Future research should examine whether the use of comparable non-EHRs platforms produces equivalent benefits to EHR use.

Our results show correlates common across disparate access and health use, while others were unique to either access or use. Older adults and individuals with less than a college degree had higher odds of belonging to classes lacking digital technologies access and to classes with fewer members engaging in health behaviors. Minority status, specifically as Hispanic or Asian American, was associated with belonging to the mobile-dependent class, consistent with available evidence.[23] Other demographics like being male and single were associated with belonging to classes with gaps in access, but were not associated with belonging to classes with limited health uses among those with access. Access to digital technologies (and skills needed for their use) is an established social determinant of health.[65,66] As digital technologies have become central to public health and healthcare, expanding access to digital technologies is a pre-requisite to engaging in various health behaviors from seeking health information online to interacting with the healthcare system electronically. Initiatives to provide Wi-Fi access during the COVID-19 pandemic could serve as a template for such efforts.[67] These efforts are critical to reduce existing disparities in access to healthcare and to preempt potential disparities emanating from digital health inequities.[36,64] Furthermore, it is important to ensure the reliability and consistency of access especially as racial/ethnic minorities have come to rely exclusively on mobile devices for internet access.[68,69] Finally, as evident in our results, single characteristics can be associated with membership of multiple classes showing near opposite access and/or use patterns. For example, people 50 + years old had higher odds of belonging to limited access/use classes (e.g., class 1) and unlimited access/moderate use (e.g., class 9) vs. class 10. This calls for examining sociodemographic profiles of class members (e.g., age and education) rather than focusing on single characteristics.

Strengths of this study include use of nationally representative data of US adults; our holistic approach to examine existing patterns of access to digital technologies and health use based on 32 behaviors; and the use of an analytic approach that allows for natural classes to emerge based on commonalities in digital technologies access and health uses, rather than forcing the data into *a priori* defined patterns. Limitations include inconsistencies in question availability, wording, and skip-logic patterns across years. For example, questions on health monitors inconsistently included examples of wearables (e.g., Fitbit), non-wearables (e.g., glucometer), or both. Access questions were seldom precise or comprehensive. For example, participants were asked whether they used broadband, a cellular data plan, or Wi-Fi to connect to the internet, but did not specify if access was at home (vs. public spaces). Thus, we used the question about whether the participant uses the internet generally as a proxy for internet access. Other limitations include the potential for different interpretations of questions, recall error, and social desirability biases typical of self-reported survey data. Accordingly, we might have misclassified people who might have had access to requisite technologies but could not or failed to report it. Some questions had specific time frames (e.g., past 12 months) while others did not. The labelling of classes (e.g., mobile-dependent) should be taken with caution because of these limitations. Many health behaviors examined here could be performed using multiple platforms. For example, sharing health information on social networking sites could be done on a website or smartphone app. However, our classification of health behaviors as web-based, mobile-based, or EHRs-based followed the question wording. Specifically, behaviors were classified as mobile-based when questions referenced smart devices or mobile features (e.g., texting) and as EHRs-based when questions referenced medical records, otherwise behaviors were classified as web-based. Finally, we could not use several covariates inconsistent over time (e.g., English proficiency). We excluded annual household income as a covariate due to high missingness.

## Conclusion

Access and use are indispensable to reaping health benefits associated with digital technologies. Seen as tools to supplement traditional health and medical care, expanding access to and health use of digital technologies has been cornerstone to national health initiatives. We showed classes of US adults with limited access to 1 + technologies and with little to no use of such technologies for health purposes. Individuals with high odds of belonging to these classes were particularly older and less educated. Patterns of digital technology access and use can shape policies and interventions targeting subpopulations among which digital health technologies are inaccessible and/or underutilized.

## Declarations

**Ethics approval.** This study only involved the use of de-identified publicly available data, which is considered "not human subjects research." "Not human subjects research" requires no Institutional Review Board review or approval per The National Institutes of Health policy and 45 CFR 46.

**Consent to participate.** Non applicable.

**Consent for publication.** Non applicable.

**Availability of data and materials.** Data are publicly available from the Health Information National Trends Survey (https://hints.cancer.gov/default.aspx).

**Competing interests.** None declared.

**Funding.** This work was supported by the Division of Intramural Research, National Institute on Minority Health and Health Disparities (ZIA MD000011).

**Acknowledgment.** The effort of PH, KK, and SEL was supported by the Division of Intramural Research, National Institute on Minority Health and Health Disparities, National Institutes of Health.

**Author Contributions.** PH ran the analyses and drafted the manuscript, DV verified the analysis, KK developed preliminary data crosswalk, SEL conceptualized the study and critically reviewed the manuscript. All authors approved the manuscript as submitted.

**Disclaimer:** The views expressed in this manuscript are those of the authors and do not necessarily represent the views of the National Institute on Minority Health and Health Disparities; the National Institutes of Health; or the US Department of Health and Human Services.

## Figures

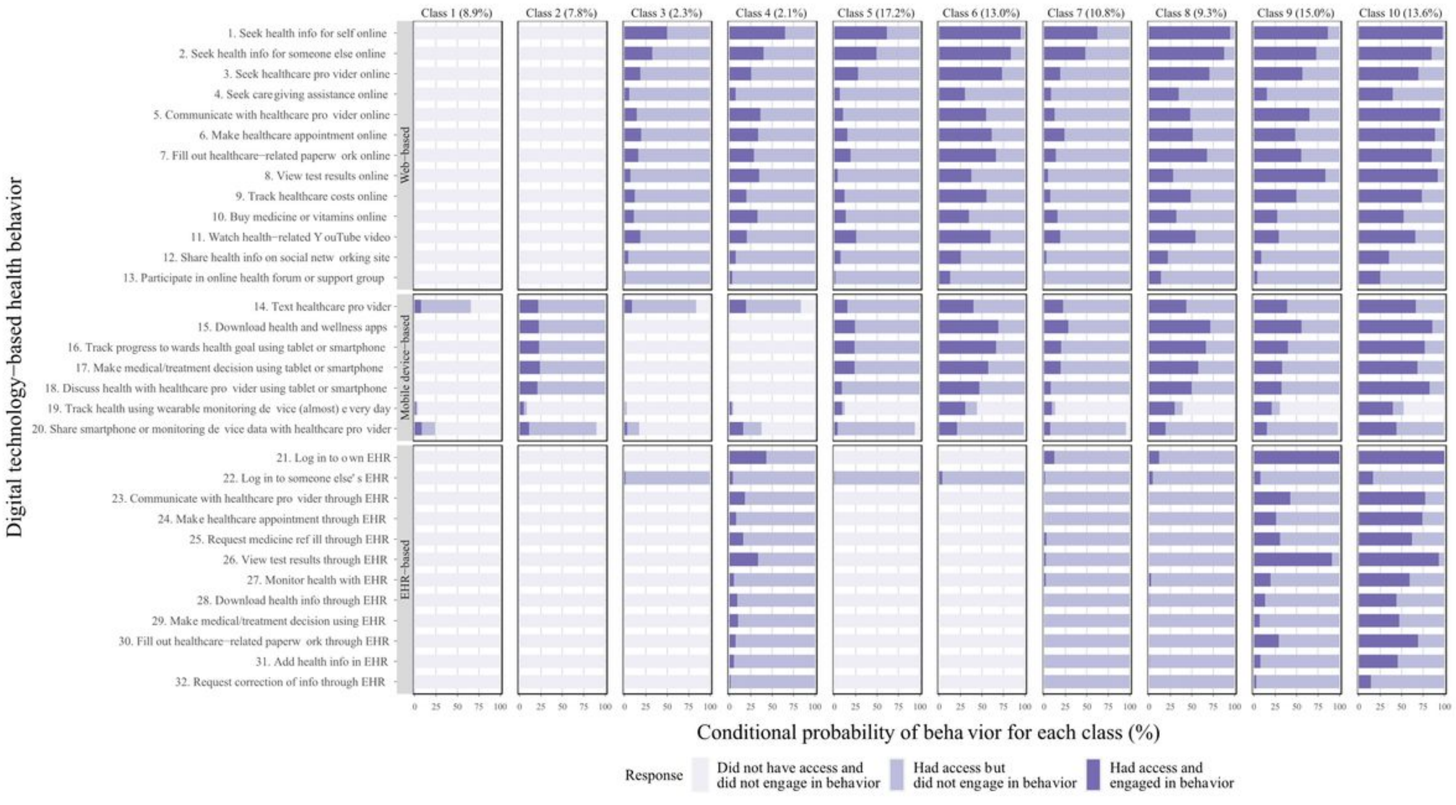

**Figure 1**

Conditional probabilities of digital technology health behavior indicator variables for the 10-class model

***Note:*** Behaviors 1 through 13 are web-based, 14 through 20 are mobile and wearable devices based, and 21 through 32 are EHR-based.

## Supplementary Files

This is a list of supplementary files associated with this preprint. Click to download.

- SupplementaryNote.docx
